# Extracellular Vesicles and Infection: From Hijacked Machinery to Therapeutic Tools

**DOI:** 10.3390/pharmaceutics15061738

**Published:** 2023-06-15

**Authors:** Diogo Gonçalves, Sandra N. Pinto, Fábio Fernandes

**Affiliations:** 1 iBB-Institute for Bioengineering and Biosciences and i4HB-Institute for Health and Bioeconomy, Instituto Superior Técnico, Universidade de Lisboa, Av. Rovisco Pais, 1049-001 Lisboa, Portugal; diogotraitolas@tecnico.ulisboa.pt; 2 Bioengineering Department, Instituto Superior Técnico, Universidade de Lisboa, Av. Rovisco Pais, 1049-001 Lisboa, Portugal

**Keywords:** exosomes, biogenesis, viral infection, bacterial infection, biofilms, therapeutic EV

## Abstract

Extracellular vesicles (EVs) comprise a broad range of secreted cell-derived membrane vesicles. Beyond their more well-characterized role in cell communication, in recent years, EVs have also been shown to play important roles during infection. Viruses can hijack the biogenesis of exosomes (which are small EVs) to promote viral spreading. Additionally, these exosomes are also important mediators in inflammation and immune responses during both bacterial and viral infections. This review summarizes these mechanisms while also describing the impact of bacterial EVs in regulating immune responses. Finally, the review also focuses on the potential and challenges of using EVs, in particular, to tackle infectious diseases.

## 1. Introduction

Extracellular vesicles (EVs) from eukaryotic cells are nano-sized vesicles. Based on their size, these structures are classified into three distinctive categories: (i) exosomes (30–150 nm); (ii) microvesicles (100–1000 nm), and (iii) apoptotic bodies (50–5000 nm) [1,2,3,4,5]. These vesicles can be produced by different biogenesis mechanisms. Microvesicles are the result of secretion to the outer media by plasma membrane budding, while exosomes have an endocytic origin. EVs are constituted by a single membrane and are secreted by all cell types, having been found in almost all body-derived fluids (e.g., urine, amniotic fluid, and plasma) [1,5]. While in the past exosomes were seen as vehicles used by cells to expel unwanted material, more recent research has found that they also participate in cell-cell communication, cell maintenance, tumor progression, stimulation of immune responses, promotion of myeline formation, neurite growth and neuronal survival [5,6,7].

EVs released from eukaryotic cells have been recognized to play a role in viral and bacterial infections [8,9]. In the context of viral infections, transmission between cells was shown to be facilitated by exosomes, as they can carry infectious nucleic acids between cells [10]. 

The physiological and pathological state of the cell is important for exosome cargo sorting. When cells are infected by viruses, the endosomal pathway is hijacked and acts to incorporate viral components into the exosomes. During a bacterial infection, the infection outcome is also impacted by the release of extracellular vesicles from the host organism. In infection caused by mycobacteria strains, exosomes were reported to be released from the human monocytic leukemia cell line THP-1 and found in the serum of infected mice [11,12,13,14,15]. These exosomes have been shown to induce the production of inflammatory cytokines TNF-α and IL-12 and to recruit both neutrophiles and macrophages in a vivo model [11,12,13,16].

Additionally, bacteria can also secrete vesicles allowing them to disperse bacterial products and thus cause potentially lethal damage to the host cells [17,18,19].

The potential use of extracellular vesicles as therapeutic nanoplatforms in the fight against viral and bacterial infections has been explored recently [9,20,21,22]. Exosomes were also demonstrated to limit virus spread in some cases. In fact, these nanoparticles were shown to contain and transport host proteins with antiviral activity, promoting resistance to infections. Thus, exosomes can be regarded both as vehicles for the transport of pathogenic material as well as protective cellular tools against infection [23,24,25]. Adding to the enormous potential of the use of EVs in clinics [26,27,28], another recent groundbreaking discovery pointed to the possibility of using EVs as decoys or scavengers of bacterial toxins [29]. 

Mechanistically, as referred to above, both viral and bacterial infections can lead to changes in the formation, secretion, and role of extracellular vesicles. This review intends then to explore and summarize the contribution of these vesicles for both viral and bacterial infections as well as to give a recent overview of the potential use of EVs as therapeutic platforms against infectious diseases. 

## 2. Biogenesis

**Figure 1:**
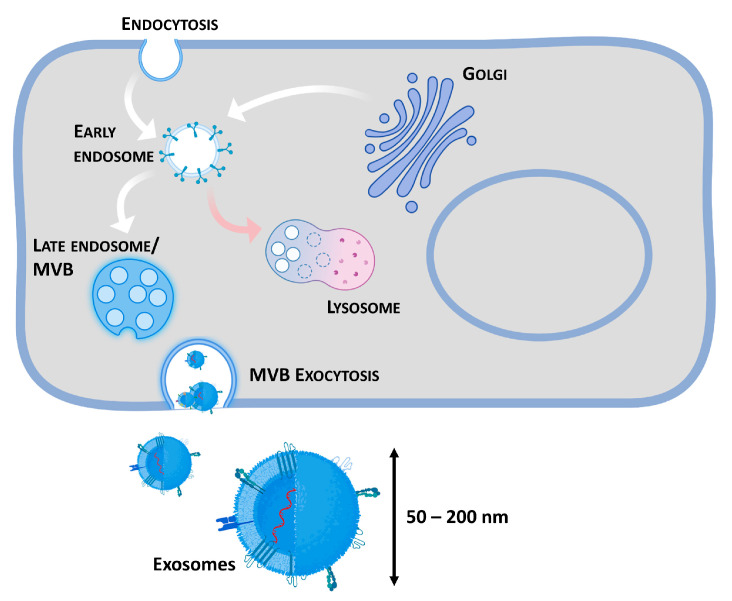
Exosome biogenesis and extracellular release. The processes of formation and release of exosomes comprise several steps: (i) formation of early endosomes from the trans-Golgi network or by endocytosis from the plasma membrane and/or; (ii) Maturation of early endosomes into late endosomes; (iii) formation of multi-vesicular bodies (MVB) through membrane invagination of late endosomes, giving rise to ILVs; (iv) MVB fusion with the plasma membrane and release of exosomes. Alternatively, lysosomes or autophagosomes can fuse with multivesicular bodies and degrade them. Created with BioRender.com.

Exosome biogenesis is naturally initiated from the endosomal pathway (Figure 1) via the invagination of the plasma membrane. In specific cases, the budding of vesicles from the trans-Golgi network (TGN) can lead to their fusion with early endosomes [30,31]. The maturation of these organelles into late endosomes and the inward budding of its membrane generates multivesicular bodies (MVB) enclosing intraluminal vesicles (ILVs). These MVBs will then fuse with the plasma membrane causing the release of the ILVs in the form of exosomes. Alternatively, MVBs can be degraded when they fuse with autophagosomes/lysosomes [30,32]. 

The formation of ILVs and MVBs requires the endosomal sorting complex required for transport (ESCRT). ESCRT 0, I, II and III, together with the accessory Vps4 complex, comprise the ESCRT and are formed by several subunits. The ESCRT machinery plays an important role in exosome secretion alongside SNARE proteins and their effectors, such as RAB GTPases [33,34,35].

The ILV-dependent ESCRT canonical pathway recruits the ESCRT complexes to the endosomal membrane via several steps. Firstly, the ESCRT-0 complex is recruited to early endosomes by the phosphatidylinositol-3-phosphate (PtdIns3P) via its HRS subunit [36,37]. HRS also recruits clathrin, restricting its clustering to microdomains and binds to ubiquitin, which is essential for sorting ubiquitinated proteins into microdomains coated with clathrin [38,39,40]. Afterward, the ESCRT-0 complex recruits the ESCRT-I complex, via the direct interaction of its HRS subunit with the TSG101 subunit of the ESCRT-I complex, to the endosomal membrane. Indeed, the absence of ESCRT-0 blocks ESCRT-1 recruitment [41,42]. ESCRT-I interacts with ESCRT-II, possibly causing endosomal membrane invagination [7]. When ESCRT-II is recruited, assembly of the ESCRT-III complex is initiated and is mediated by the direct binding of CHMP6 to EAP20 [43,44,45]. The complex is required for the breakage of ILV into the MVB lumen. Other non-canonical pathways are also able to support ILV biogenesis [32,46]. Lastly, MVBs can either fuse with lysosomes exposing ILVs for degradation or can fuse back with the plasma membrane, releasing ILVs into extracellular space as exosomes. This last step is performed by the SNARE proteins. The role of these proteins in membrane fusion has been intensively explored [47,48,49].

Microvesicles are vesicles that are formed by the direct outwards budding of the plasma membrane of cells. Their formation is thought to require cytoskeleton components (such as microtubules or actin), molecular motors, SNAREs, and tethering factors. This mechanism is less well characterized but also involves the ESCRT machinery [32,50,51]. One of the mechanisms of microvesicle biogenesis requires the recruitment of subunits TSG101 and VPS4 of the ESCRT complex to the plasma membrane via the adaptor protein arrestin domain-containing protein 1, thus promoting the generation of microvesicles [52].

Small GTPase proteins that include RhoA, ARF1 and ARF6 also have the capability to promote the formation of extracellular vesicles through outwards budding from the plasma membrane of cancer cells, forming microvesicles [53,54,55]. Furthermore, the activation of the member of the sphingomyelinase family, acid sphingomyelinase, on the plasma membrane via the generation of ceramides can cause the release of microvesicles from red blood cells and glial cells [56,57].

Apoptotic bodies are released upon cell death into the extracellular space and are the result of the separation of the plasma membrane from the cytoskeleton because of hydrostatic pressure [58,59]. The apoptotic body formation is a critical process of cell death; these vesicles can vary in size and can contain a variety of cellular components such as degraded proteins, DNA fragments, chromatin remnants, micronuclei, and intact organelles. Apoptotic bodies, after formation, are usually degraded within phagolysosomes from macrophages, parenchymal cells, or neoplastic cells. Inflammatory responses aren’t associated with apoptotic bodies since they do not release free cellular constituents into the surrounding tissues [60].

## 3. Function and Composition of EVs

EVs have various physiological and pathological roles in cells [1,30,61]. The exosome protein sorting pathway can be used by cells to regulate anterior-posterior cell polarity [62]. The anterior-posterior cell polarity is utilized by rapidly migrating cells such as human leukocytes, and, unlike epithelial cell polarity (that develops in long time scales), it occurs within minutes of contact with polarizing stimuli. Particularly, this exosome pathway creates a polarized protein sorting pathway that increases exosome-associated proteins and lipids at the posterior pole of amoeboid cells. Nevertheless, despite these mechanisms not being fully elucidated, their presence allows leukocytes and other amoeboid cells to rapidly generate the polarity needed for cell migration [63].

When released from cells, EVs can be integrated into components of the extracellular matrix. There, EVs offer a mechanism that allows cells to manipulate the extracellular matrix composition [62]. The modulation of the extracellular matrices by EVs has also been associated with the formation, development, and propagation of amyloid aggregates in neurodegenerative diseases [61]. In this context, EVs were shown to contribute to the propagation of Aβ42 or tau proteins, which could play a role in Alzheimer’s [64,65], while also transporting Fas-associated factor 1 and α-synuclein oligomers, which are associated with Parkinson's disease [66,67].

Additionally, EVs play a role in the transmission of signals and molecules via intercellular vesicle pathways and have the capability of delivering multiplexed, combinatorial signals via clustering and contact of cognate receptors on the cell surface during cell-cell communication [62]. 

Through time, exosomes have been discovered in almost every single body fluid. They showed specific profiles of proteins, miRs and lipids that can mirror the origin and physiological state of the cell they came from so that they can be used as biomarkers of various diseases [68,69,70,71,72,73]. Although biological fluids rich in exosomes can be easily acquired, the actual use of exosomes as biomarkers is not yet common clinical practice [74].

Due to the ability of exosomes to target specific cells or tissues, they are able to mediate the horizontal transfer of cargo via the interaction of surface adhesion proteins. Recently, several studies have revealed that exosomes can be used as therapeutic vehicles for the delivery of drugs or biological therapeutics across different targeted cells [75,76,77,78].

Microvesicles are also involved in cell-cell communication between local and distant cells. For instance, they have been documented to encompass a grand variation of molecular cargo. This cargo reflects the cell type from which the microvesicle was released. Microvesicle cargo can include proteins such as active proteases, integrin receptors, small GTPases and even miRNA processing machinery. They are also known to include nucleic acids, ROS regulators and active lipids [79].

Although the mechanisms that comprise the biological effects of microvesicles and their active molecules remain unclear, it has been proven that they can participate in the pathophysiological process of different organs by transporting cargo such as proteins, lipids, and microRNAs. The interaction of these vesicles with cells has been confirmed and shown to regulate autophagy through the PI3K-AKT pathway, apoptosis through the Fas/FasL pathway, and inflammation through the NF-kB pathway [80]. 

**Figure 2:**
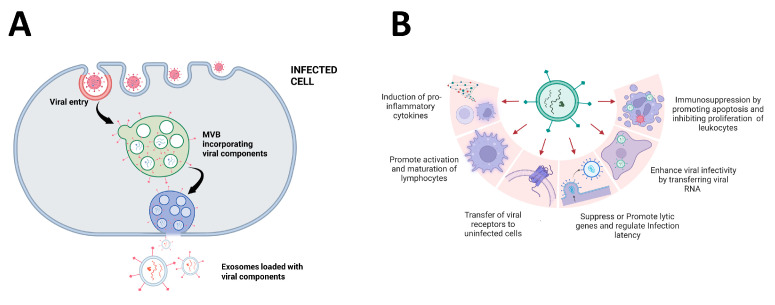
Potential effects of EVs in viral infections. (**A**) Incorporation of viral components in exosomes released from infected cells. (**B**) EVs from infected host cells have been reported to induce both the promotion and inhibition of viral infection. The figure describes some of these reported effects as described in the text. Created with BioRender.com.

The components of EVs differ in cell type/origin, but, in general, eukaryotic-derived EVs are enriched in nucleic acids, proteins, lipids and glycoconjugates (Figure 2) [81]. The most abundant lipid classes found in EVs are sterols (cholesterol), sphingolipids and glycerophospholipids [81]. Interestingly, exosomes exhibit higher phosphatidylserine levels than multivesicular bodies and apoptotic bodies [82]. EVs also carry a great diversity of genetic material such as DNA, mRNA, mitochondrial RNA, microRNAs (miRNAs), long intergenic non-coding RNAs (lincRNAs) and circular RNAs (circRNAs) [32]. In terms of protein composition, several proteins commonly found within exosomes have been identified, and many of them are recognized as EV markers [83,84]. These include (i) transmembrane proteins—tetraspanins (CD60, CD63, CD9, CD81 and others), integrins, cell adhesion molecules, growth factor receptors, heterotrimeric G proteins (GNA) and phosphatidylserine-binding MFGE8/lactadherin (ii) cytosolic signaling molecules (CD55, CD59, CD82, and Rabs), intracellular proteins including heat shock proteins (HSP70 and HSP90), and extracellular proteins such as fibronectin, collagen, cytokines, growth factors, and matrix metalloproteinases. The protein content in EVs is known to facilitate the deliverance of cargo [9,81,85,86]. Tetraspanins (CD63, CD81, and CD9) are present in both healthy and pathology associated-cells [86].

## 4. Promotion of Viral Entry and Replication

Extracellular vesicles, and in particular exosomes, have been found to have potential antagonistic effects in viral infections, assisting in propagating viruses while simultaneously mediating antiviral responses (Figure 2) [87]. In fact, EVs can confine viral genomes and transfer them to susceptible cells, aiding their spread and immunity evasion. On the other hand, they can also transfer antiviral elements, assisting cells in the activation of antiviral mechanisms by the host organism [88,89]. 

Exosomes have similar structural and molecular features to certain viruses (e.g., HIV-1 and HIV-2), as they are covered by a lipid bilayer that contains RNA [90], lipids [91], proteins [92,93], and carbohydrates [94], in addition to having similar dimensions [84] and buoyant density ranging from 1.15 to 1.21 g/mL [95]. These similarities can explain, in part, the different roles of exosomes during viral infection (Figure 2) [96]. Virion assembly is closely related to virus budding; thus, most viruses use their own structural proteins to recruit the endosomal sorting complexes required for transport (ESCRT) machinery [97,98]. Examples of this are oligodendrocytes that, through the ceramide machinery pathway, begin exosome formation, while other types of cells use tetraspanin complexes [99,100,101].

Instead of hijacking the ESCRT pathway, certain viruses have been found to exploit the Rab GTPase complexes for replication and release purposes. The RSV, Hantavirus, and IAV have all been found to exploit the Rab pathway for transport trough the plasma membrane [102,103,104,105]. Additionally, if interference occurs with the Rab11 protein levels, it can cause a promotion or inhibition in the release of EVs that carry different compounds, such as transferrin, anthrax toxin, HSP-70, and flotillin [102,106,107]. Rab27a, another member of the Rab family, was shown to be an important component for exosome biogenesis during MVB fusion with the plasma membrane [108,109]. The levels of Rab27a were shown to increase with the presence of cytomegalovirus infection [110]. HIV1- proteins also interact with Rab27a and have been proven to promote the formation of exosomes [111,112]. The association between tetraspanins and the Gag protein from HIV-1 indicates that the assembly and release of HIV-1 involve lipid raft domains that are rich in these tetraspanins, such as CD63 and CD81 [113]. HHV-6, the Human Herpesvirus 6, promotes the formation of MVBs that aid in the survivability and release of the virion together with exosomes, indicating exploitation of the same pathway [114]. The HHV-6 glycoprotein gB was found to have an association with CD63, cementing the importance of the endosomal pathway in the infection and assembly of HHV-6 [114].

Blocking the release of EVs suppressed in vitro Hepatitis C virus (HCV) replication [115]. HCV can, in this way, use EVs as a tool for its transmission and infection. Similarly, hepatocytes infected with HCV were shown to excrete EVs with the entire HCV genome while also being able to transfer RNA from HCV, leading to viral replication and infection in the host cells [10,116]. The Hepatitis G virus can employ EVs to transport viral RNA to cells, such as mononuclear peripheral blood cells, to form a basis for infection [117]. 

Specific proteins and other viral components were found to be crucial for viral entry and replication into host cells. This is the case of SARS-CoV-2, its spike (S) protein located at the virus’s surface was shown to bind to angiotensin-converting enzyme 2 (ACE2) of host cells, and this step is essential for promoting the viral entry [118]. EVs containing ACE2 were then shown to prevent infection caused by SARS-CoV-2 Spike protein, and thus blocking one of the initial infection steps through this interaction could be a viable therapeutic strategy against coronavirus [119]. 

Also, viral RNA, such as TAR RNA, was detected in exosomes isolated from HIV-1 patient samples. TAR RNA is known to down-regulate apoptosis, and thus host cells are more susceptible to enhanced HIV replication [120]. It was also revealed that HIV-1 replication could be hampered through the use of the catalytic subunit 3G of the editing enzyme of apolipoprotein B mRNA, which is a cytidine deaminase that contributes to the antiviral cellular response against retroviruses by the accumulation of exosomes in cells adjacent to one another. After fusing with dendritic cells, HIV-1 sorts antigens in exosome-like vesicles, and it was shown that exosomes could promote viral infection by transporting viral antigens and transferring their cargos to cells [89,121,122]. Examples of viruses utilizing the vesicular trafficking machinery include the exosomal tetraspanins CD81 and CD63 that assist the virion by interacting with HIV-1 Gag and the Human herpesvirus 6 virions that use the same pathway of formation of extracellular vesicles and exosomal release pathway [114].

Human herpesvirus, particularly KSV and EBV, have been described to be capable of recruiting the exosome pathway to release several components that include miRNAs, mRNAs, small non-coding RNAs, and proteins [123,124,125,126]. For instance, cells infected with EBV release exosomes that contain viral miRNAs believed to be smaller products of the transcripts of BamH1 EBV. These work in association with cellular miRNAs to induce an expression of target genes by modulating recipient host cells [127,128,129,130]. 

Oxidative stress can also play a role in the EV-Viral replication relationship, as it was already hypothesized that EVs from cervical cancer cells carrying oxidative stress factors might have the potential to exacerbate HIV-1 replication in HIV-1-infected macrophages [131] via ROS generation.

EVs secreted by a host during infection have also been shown to carry infectious material to a second host. As an example, Zika virus-infected mosquito cells secrete EVs that were demonstrated to mediate infection between mosquito and mammalian cells [132].

Interestingly, virus-associated EVs are often enriched in the negatively charged glycerophospholipid phosphatidylserine (PS). In healthy cells, PS is present in the inner plasma membrane leaflet, and its translocation to the outer leaflet is often associated with apoptosis and cell stress. This exposed PS is regarded as an evolutionarily-conserved signal that it is both immunosuppressive and anti-inflammatory [133]. External PS has been able to create infection latency when usurped by viruses, microorganisms, and parasites [134]. 

## 5. EVs in Bacterial Infection

The antibacterial character of host EVs has been described in different studies [135,136]. The production of antibacterial EVs by neutrophils was observed in response to S. aureus infection. This was due to the stimulation of the complement receptors Mac-1. Since spontaneous EV formation is independent of Mac-1 signaling, the production of EVs in response to infection is likely to be associated with a different pathway [137]. 

The exact mechanisms involved in the stimulation of EV production during bacterial infection have been partially identified in some cases. During infection, bacterial cells can be shuttled to MVBs and finally to lysosomes, where pathogens are degraded. In bladder epithelial cells (BECs) infected with uropathogenic *Escherichia coli* (UPEC)*,* this pathogen was shown to neutralize lysosome function. This neutralization of lysosomes triggered in BECs the fusion of this organelle directly with the plasma membrane and led to the expulsion of UPEC encased in large EVs. This encasing is likely to be the result of bacterial stopover in MVBs, and it may also be a strategy to reduce the reattachment of expelled bacteria to the bladder epithelium, ensuring elimination in urine [138].

**Figure 3:**
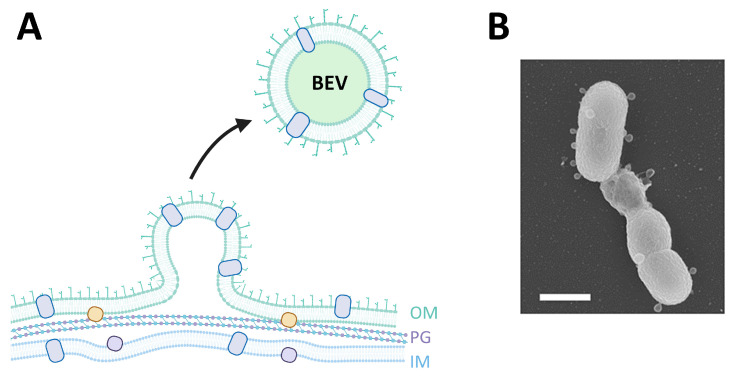
Biogenesis of bacterial extracellular vesicles (BEV) by blebbing of the outer membrane (**A**) and SEM visualization (**B**); the diameter of these vesicles is commonly smaller than 400 nm. OM: outer membrane, PG: peptidoglycan, IM: Inner membrane. Figure (**B**) shows a Pseudomonas *aeruginosa strain*, PAO1 mutant deficient in Opr86, producing several extracellular vesicles. The scale bar corresponds to 1 µm. Figure (**B**) is reprinted with permission from Ref. [143] Copyright 2011 *Environmental Microbiology*.

Importantly, EV production is not an exclusive process of eukaryotic cells, as bacteria are also capable of releasing extracellular vesicles, descriptively named bacterial extracellular vesicles (BEVs). The diameter of these vesicles is commonly smaller than 400 nm [139] (Figure 3). BEVs are obtained from different pathways and can play different roles [140,141]. In general, BEVs from gram-negative bacteria are produced from the blebbing of bacterial outer membrane or via explosive cell lysis (Figure 3). The main components are LPS, as well as proteins (such as OmpA) and phospholipids [140]. Gram-positive BEVs are produced via endolysin-triggered bubbling cell death [142] and can contain LTA, while other components are similar to those commonly found in BEVs obtained from gram-negative bacteria. Additionally, BEVs from both cell types present DNA, RNA, and virulence factors [142].

BEVs were immunogenic in animal models and were also shown to produce strong physiological and molecular changes in the host [17,19]. Immunogenic responses can be provoked from BEVs isolated from pathogenic bacterial sources and from BEVs from symbiotic or commensal bacterial. This is the case of vesicles isolated from culture supernatants of the pathogenic *Bacillus anthracis*, a gram-positive bacterium [17]. These vesicles were shown to be potent vehicles for toxin transmission to host cells, causing a significant reduction of viability in macrophage monolayers. EVs from pathogenic bacteria and EVs isolated from infected organisms were also shown to trigger an immune response that, in extreme cases, can result in sepsis [144,145,146]. In contrast, BEVs isolated from the gut bacterium *Akkermansia muciniphila* (commensal bacteria) were shown the ability to down-regulate the production of IL-6 from colon epithelial cells, thus possibly conferring protection during colitis [147].

In the last decade, BEVs have also been described as having a potential effect on biofilm formation [148]. Biofilm formation is a major concern in healthcare, being associated with several human pathologies, such as the non-healing status of chronic wounds and cystic fibrosis [149]. Once established, biofilm infections are extremely challenging to eradicate as they are reported to be much more resistant to antibiotics than planktonic bacteria (free-swimming state) [150]. After initial invasion, bacteria can attach to living and medical surfaces and form a densely-packed 3D biofilm structure protected by a self-generated extracellular polymeric substance (EPS) matrix. This is constituted by polysaccharides, lipids, proteins, and nucleic acids [151]. Many proteins found in biofilm matrix have been associated with biofilm EVs [143,152], but the biological role of BiEVs (biofilm EVs) is still not yet fully understood. In *P. aeruginosa* biofilms, many proteins found in BiEVs are related to iron acquisition [152], which is relevant since iron in high concentration can promote the transition between planktonic to biofilm state [153]. 

BiEVs are also able to scavenge antibiotics such as gentamycin [154], thus acting as decoys of antimicrobial agents. Additionally, the presence of BiEVs appears to mediate the initial bacterial adhesion and invasion steps [155]. 

## 6. Impact of EVs on the Immune System

Virus-infected cells are capable of releasing EVs that export viral components such as small RNA and proteins (Figure 2A). The viral components on the cargo of EVs are ultimately responsible for the pathophysiological effects on the target cells [126,156]. Virus-infected cells shed EVs that can induce immune evasion, cytokine modulation, apoptosis, transcellular spread, and proliferation (Figure 2B) [157,158]. These effects have the potential to modulate the immune responses of the infected organisms.

EVs prevenient from cells infected with the Ebola virus enhanced its activity and capacity for infection. The cargo of these EVs was shown to contain cytokines that contributed to the virus pathology, as well as VP40, a matrix protein from the same virus. These components contributed to a decrease in the viability of T cells and monocytes [159,160].

The downregulation of the lytic gene expression can be exploited by some viruses to help escape from immune responses in infected cells since the triggering of the immune system is reliant on the expression of viral antigens [161,162]. Studies have shown that HSV-1 infection is accompanied by the export of several viral components within exosomes, including microRNAs which reduce the synthesis of viral components, curtailing the spread of cellular infection as a strategy to moderate more aggressive symptoms and promoting the spread between individuals. [163]. Possibly with the same goal of curtailing the spread of infection and promoting person-to-person spread, HSV-1-infected cells were also shown to secrete exosomes loaded with STING, an innate immune system activator [164]. STING was also shown to be stimulated by host EVs containing bacterial DNA from *Listeria*, *Francisella tularensis*, and *Legionella pneumophila* [165,166]. In the case of *Listeria L. monocytogene* infection, the activation of STING caused a distinct response depending on the cell type. For T cells, it led to the activation of apoptosis, while an increase in IFN-β production was observed in infected macrophages [166]. 

The protein APOBEC3G (a cytidine deaminase) [167] can inhibit viral replication via G-to-A mutations in the transcribed viral DNA [168]. APOBEC3G has been shown to be secreted by cells in exosomes that confer resistance to HIV-1 in exosome recipient cells.

Serum samples collected from patients with chronic gastritis and positive for *Helicobacter pylori* revealed the presence of exosomes. These exosomes from infected patients were shown to have a stimulating effect on the IL-6 receptor located in human gastric epithelial cells, resulting in the release of pro-inflammatory cytokine IL1-α, hinting at the role of exosomes in triggering immunological responses during *H. pylori* infection [169].

EVs, beyond their role as vehicles for the transfer of viral components and inhibitors of the immune system, can also trigger an immune response through the incorporation of specific virus-encoded proteins and nucleic acids [88,89,170].

Carvalho et al. demonstrated that T cells were able to produce EVs containing CD4 receptors, which act as HIV-1 decoys during infection. HIV-1 infected cells were also shown to secrete Nef-loaded exosomes, which depleted CD4 levels, confirming the complexity of the roles of exosomes in infection as they can be employed both as hijacked viral agents and as part of host innate defense mechanisms [171]. 

Both *Mycobacterium tuberculosis* and *Mycobacterium avium* are intracellular bacteria that can survive on the host macrophage phagosome. During infection caused by mycobacteria strains, exosomes were shown to be released from monocytic leukemia (THP-1), monocyte and macrophage cells (Raw264.7) in vitro models [11,12,14]. Exosomes released from the infected beforementioned cells after a Mycobacteria infection were shown to induce TNF-α and IL-2 production [11,12]. Interestingly, treatment with exosomes released from cells infected with *Mycobacterium tuberculosis* can promote macrophage recruitment and thus stimulate the immune response of the host [11]. Moreover, EVs released from infected cells during infection with mycobacteria were shown to activate antigen-specific T cells in vivo (CD4^+^ and CD8^+^) [172]. Mycobacterial RNA within released EVs also improves the maturation of phagosomes, which results in antibacterial immunity in the recipient cells [172].

In the same context, macrophages infected with salmonella have been reported to release exosomes that showed pro-inflammatory capabilities, with TNF-α being produced in human monocytes. These exosomes were shown to contain LPS, a component of the cell wall of gram-negative bacteria [173]. T-cell inactivation was also shown to be induced by mycoplasmas that infect tumor cells, as their exosomes induce the production of anti-inflammatory IL-10 in B cells [174].

EVs have been suggested to contribute to dendritic cell maturation during infection with *Chlamydia muridarum* [9]. Infection of dendritic cells by *Chlamydia psittaci* was characterized by an increase in the release of exosomes that promoted the production of IFN-γ from natural killer cells, contributing to the protective immunity against *Chlamydia* [175]. *Chlamydia trachomatis* DUF582 protein was reported to interact with proteins from the host, such as Hrs and TSG101, that are involved in EV biogenesis [176]. Nevertheless, inhibition of the expression of these proteins did not have a negative impact on chlamydial growth in HeLa and HEK-293 cell culture models. It is important to note that the studies described here with chlamydiae were performed in vitro, and the potential of EVs in more relevant models of fungal infection is yet unexplored [176].

## 7. The Potential of EVs to Tackle Viral and Bacterial Infections

EVs present several innate advantageous properties as therapeutic vehicles, such as their ability to cross biological barriers such as the blood-brain barrier, their long circulating half/life and their immunostimulatory efficiency [177,178]. For this reason, their potential use as therapeutic platforms is being strongly pursued. Potential applications range from exploring their use in immunotherapy, diagnostic markers, drug delivery systems and as tools against targeting hereditary diseases [178,179].

EVs have been considered as potential carriers of biomarkers for human diseases or to be themselves relevant biomarkers [180]. For instance, they have been found in cerebrospinal fluid containing alpha-synuclein (which is a protein associated with Parkinson’s disease) and as biomarkers for tumors and cancer (such as glioblastoma) [61,180,181,182,183]. Their use as biomarkers is facilitated since these vesicles are present in several human fluids, including blood and urine, thus allowing minimally or non-invasive biopsies that can be used to monitor or diagnose a potential disease [81].

Another strategy involves the use of EVs for viral immunization. The key domain for coronavirus recognition and EVs presenting coronavirus spike S protein has been suggested to be used as vaccines against coronavirus. The incorporation of a small domain or full-length S protein in EVs has been demonstrated for both SARS SARS-CoV-1 and SARS-CoV-2 [184,185]. Presentation of the viral protein is achieved through the fusion of the protein with vesicular stomatitis virus glycoprotein G (VSG-G), resulting in the incorporation of that protein in small EVs. Similarly, ginger exosome nanoparticles (GLEN) collected from the root ginger showed a protective effect against SARS-CoV-2 [186]. 

In the context of the recent SARS-CoV-2 pandemic, a large number of EV-based formulations have undergone or are currently undergoing tests in clinical trials [187]. A selection of these trials is listed in Table 1, reflecting the high expectations for EV-based therapeutics in infection. On the other hand, EVs released after stimulation of the leukemic monocyte cellular line THP-1 LPS trigger the release of cytokines, such as IL-1β, CCL5, C-C motif, and TNF-α, thus modulating the inflammatory response in mice. In fact, these exosomes have been shown to be useful as adjuvants in hepatitis B vaccination strategies, as they enhance the cellular immune response in mice [188].

Engineered exosomes can also be utilized as vehicles for targeted drug delivery in antiviral therapeutics. As previously noted, the HIV-1 Nef adaptor protein is often found in exosomes, having multiple roles in viral replication and pathogenesis [189]. Given the ability of Nef to accumulate in multivesicular bodies, the fusion between viral antigens and a mutant of the exosome anchoring HIV-1 Nef ultimately leads to the production of immunogenic exosomes [190]. This Nef-based engineering strategy was reported to generate exosomes for immunization against the hepatitis B virus (HBV) via cytotoxic T lymphocytes [190]. The authors of that study hypothesized that the constructs could be used for therapies developed against AIDS and hepatitis B.

Exosomes collected from HIV-1 patients showed that viral elements are eliminated via these vesicles trough excretion. Breast milk [191] and semen [88,192] derived exosomes from healthy individuals have been shown to moderate HIV-1 infections in vitro, suggesting that these particles could be employed in potential therapeutics for AIDS. 

**Figure 4:**
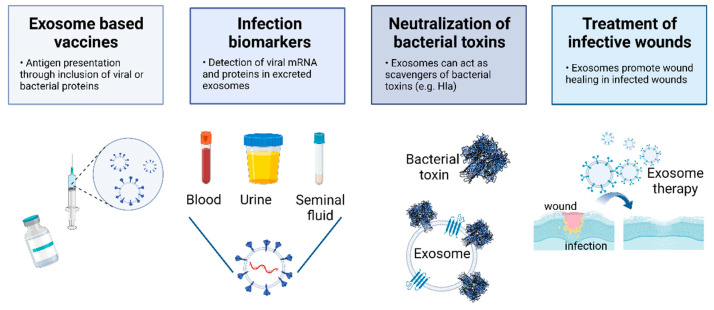
Possible uses of EVs as therapeutic or diagnostic tools in infection diseases. Created with BioRender.com.

Concerning bacterial infections, EVs were recently shown to constitute an innate immune response by neutralizing bacterial toxins from Methicillin-resistant *Staphylococcus aureus* (MRSA). MRSA infections are commonly associated with high rates of mortality, and the production of bacterial pore-forming toxins (PFT) plays an important role in MRSA pathogenicity [193,194]. Intravenous injection of heat-killed *S. aureus* into mice led to an increase in the number of exosomes in the blood stream [29]. Further testing revealed that vesicles harvested from mice in contact with heat-killed *S. aureus* offered protection to the A549 cell line against alpha-hemolysin. Importantly, the authors found that exosomes from cells overexpressing a disintegrin and metalloprotease 10 (ADAM10), an alpha-hemolysin receptor, conferred a higher degree of protection to the in vitro cell model (Figure 4). On the other hand, exosomes isolated from ADAM10 KO cells offered no cellular protection against bacterial infection. These results clearly offer new possibilities in the combat against antimicrobial-resistant diseases. One promising strategy is likely to be the use of EVs with neutralizing activity against bacterial toxins in combination with traditional antibiotics [29].

The combination of EVs with other nanomaterials has also been demonstrated. Chitosan-Silk Fibroin dressing containing both silver nanoparticles and host EVs increased the rates of wound healing in a *P. aeruginosa*-infected mouse skin wound defect model [195]. 

Another potential use of EVs is the preparation of vaccine platforms. In this sense, BEVs are the top contenders to be used in vaccines against the respiratory pathogen *Streptococcus pneumoniae* [196]. This is because these bacterial nanovesicles can carry crucial antigenic molecules with much less virulence for the host organism. 

Figure 4 summarizes different avenues for the use of EVs to tackle viral and bacterial infections.

## 8. Conclusions

While the exact mechanisms are not fully understood, many viruses have been shown to hijack the exosome pathway machinery to produce both viral particles and EVs loaded with viral components that can infect other cells. These particles were also recently found to play crucial roles in inflammation and immunomodulation during infection. Thus, they present potential both for increasing the pathogenicity of infectious agents as well as for acting as promoters of immunogenicity. These roles of EVs during infection suggest many potential avenues for therapeutics. Likewise, bacterial extracellular vesicles (BEVs) were also shown to play a dual role during infection as they can be potent vehicles for toxin transmission to host cells while also strongly contributing to immunogenic responses. Furthermore, the role of BEVs in triggering biofilm formation has also been identified, confirming the importance of these structures. 

While further research is necessary to fully comprehend the potential of exosome-based therapeutics in infection, EVs have already been shown to be both effective therapeutic tools and diagnostic biomarkers for infections. Several significant hurdles remain in the path of exosome-based therapeutics, namely the difficulties in standardization and large-scale isolation. While precipitation-based methods allow for high-yield EV isolation, they do so at the cost of sample contamination with other precipitants [197]. Immunoaffinity methods solve the issue of specificity and allow for the recovery of very pure samples, but scaling and maintenance of functionality are impacted. Filtration-based methods offer a balance between purity, functionality and yield. Recent innovations in tangential flow filtration allowed for an increase in the efficiency of EV isolation. On the other hand, the implementation of microfluidic-based isolation has the potential to facilitate the standardization and reproducibility of procedures [198]. Given the complexity and novelty of EVs as therapeutics tools, one further problem is the absence of regulatory frameworks for their use. Solving these issues is now the major challenge and future therapeutic applications of EVs will have to address them.

## Figures and Tables

**Table 1:** List of completed or ongoing trials aiming to test the safety and effectiveness of EVs in COVID-19-associated illnesses. Bone-marrow mesenchymal stem cells (BMSC). Amniotic-fluid mesenchymal stem cells (AFMSC). Adipose tissue mesenchymal stem cells (ADMSC) Umbilical-cord mesenchymal stem cells (UCMSC). Perinatal mesenchymal stem cells (PMSC) Acute Respiratory Distress Syndrome (ARDS). * Recruiting or active trials.

Formulation Name	EV Origin	Therapeutic Application	Clinical Trial Identifier
ExoFlo™	BMMSC	Moderate-to-severe ARDS in patients with COVID-19	NCT04493242 NCT04657458 *
ExoFlo™	BMMSC	Post-Acute COVID-19 and chronic Post-COVID-19 syndrome.	NCT05116761 *
UCMSC-EV	UCMSC	COVID-19 pneumonia	NCT05787288 *
UCMSC-EV	UCMSC	Chronic cough after COVID-19 infection.	NCT05808400 *
EV-Pure™ and WJ-Pure™	Placenta and UCMSC	Moderate or severe ARDS in patients with COVID-19	NCT05387278
Zofin™	AFMSC	Treating COVID-19 long haulers	NCT04384445 *
Zofin™	AFMSC	Patients with mild to moderate COVID-19	NCT04657406 *
Zofin™	AFMSC		NCT05228899 *
CSTC-Exo	COVID-19-specific T cells of convalescent patients	Early-stage COVID-19 pneumonia	NCT04389385
haMSC-Exos	ADMSC	Severe COVID-19-related pneumonia	NCT04276987
EXO-CD24	CD24 expressing 293-TREx™ derived from HEK-293 cells	Moderate or severe COVID-19 infection	NCT04747574
EXO-CD24	CD24 expressing 293-TREx™ derived from HEK-293 cells	Moderate or severe COVID-19 infection	NCT04969172
EXO-CD24	CD24 expressing 293-TREx™ derived from HEK-293 cells	Moderate or severe COVID-19 infection	NCT04902183 *
ARDOXSO	PMSC	ARDS or COVID-19 pneumonia	NCT04798716 *
MSC-Exosomes	MSC	Immune modulation in COVID-19 infection	NCT05191381 *

## Data Availability

Not applicable.
